# Asiatic Cholera in Virginia

**Published:** 1866-05

**Authors:** 


Asiatic Cholera in Virginia.—It is a noteworthy and astonishing fact that, during the forty years in which this scourge has afflicted the earth, no case of Asiatic cholera has ever occurred in the basin of country embracing the mineral springs of Virginia.—*London Lancet.*

